# Multirefractory bullous pemphigoid, psoriasis and psoriatic arthritis successfully treated with guselkumab^☆^

**DOI:** 10.1016/j.abd.2023.07.017

**Published:** 2024-06-13

**Authors:** Francisco José Rodríguez-Cuadrado, Gaston Roustan-Gullón, Dolores Suárez-Massa, Mercedes Hospital-Gil

**Affiliations:** a Department of Dermatology, Hospital Universitario Puerta de Hierro Majadahonda, Majadahonda, Spain; b Department of Pathology, Hospital Universitario Puerta de Hierro Majadahonda, Majadahonda, Spain

Dear Editor,

A 48-year-old man who had been followed for 12 years for moderate plaque psoriasis only treated with topical corticosteroids, such as mometasone furoate and betamethasone dipropionate (he had never received systemic treatment or phototherapy), presented with an outbreak of pruritus and pruritic blistering lesions and a severe worsening of his psoriasis (Fig. 1, Fig. 2). The biopsy of these lesions showed subepidermal blisters with eosinophilic inflammatory content (Fig. 3A), and the direct immunofluorescence test demonstrated linear deposition of C3 (Agilent Dako, reference F0201) and IgG (Agilent Dako, reference F0202) in the basement membrane (Fig. 3B and 3C). These findings, together with the ELISA (enzyme-linked immunosorbent assay) positivity for anti-basement membrane zone antibodies (IgG antiBP180 28 RU/mL, IgG antiBP230 33 RU/mL; ARUP Laboratories, test code 0092566) confirmed bullous pemphigoid (BP).

**Fig. 1:**
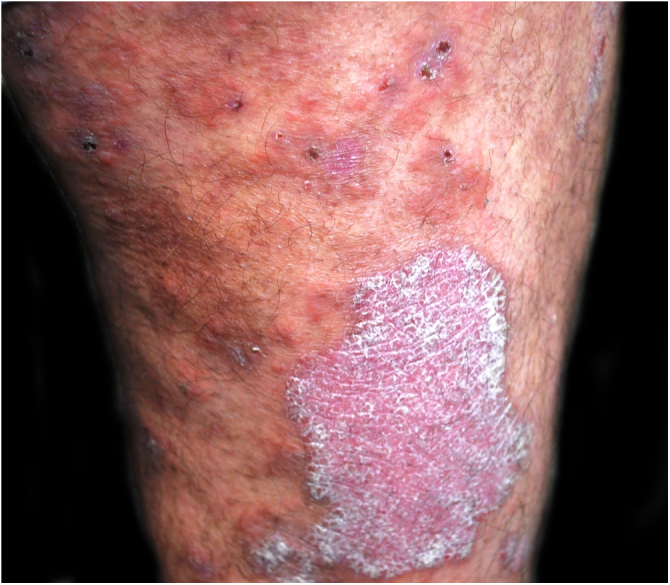
Plaque psoriasis on the thigh. In addition, some erosions are observed as a result of broken pemphigoid blisters.

**Fig. 2:**
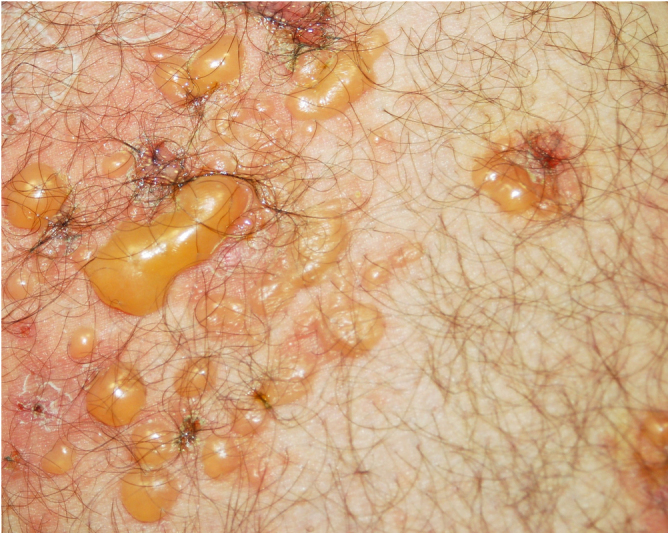
Tense blisters with erythematous base.

**Fig. 3:**
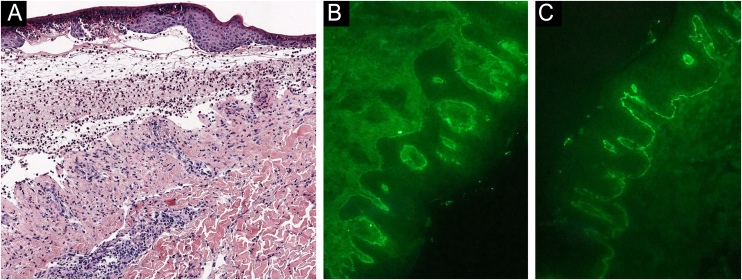
Biopsy of the blisters. (A) Subepidermal blisters with eosinophilic inflammatory content (Hematoxylin & eosin stain). (B) Linear deposition of C3 in the basement membrane (direct immunofluorescence). (C) Linear deposition of IgG in the basement membrane (direct immunofluorescence).

Thus, he started azathioprine 100 mg/day with little response after 11 months of treatment. The change to cyclosporine 300 mg/day was initially effective but failed to achieve adequate control 13 months later. The initiation of methotrexate 7.5 mg weekly did achieve almost complete remission of both pathologies within 2-months. However, it had to be discontinued in the third month after its initiation due to persistent diarrhea and thrombocytopenia.

Subsequently, etanercept 50 mg weekly was started due to the onset of psoriatic arthritis. The response was good for 6 years, but a chondroid neoplasm was identified which, although it seemed to be an enchondroma, suspension of the drug and close follow-up were recommended. This led to the exacerbation of BP and the worsening of psoriasis.

A combination of omalizumab 300 mg monthly and doxycycline 100 mg/day was then prescribed, but the BP was not controlled, and the psoriasis worsened to Psoriasis Area and Severity Score (PASI) 10 and Dermatology Life Quality Index (DLQI) 15, so after 7-months it was decided to switch to guselkumab monotherapy: 100 mg by subcutaneous injection at weeks 0 and 4, followed by a maintenance dose every 8-weeks. One month after the first dose, the patient presented PASI0, and one month later the BP lesions disappeared (Fig. 4). To date, 2-years after the initiation, he continues with guselkumab 100 mg every 8 weeks, and remains with PASI0, with no radiologic signs or symptoms of arthritis (Psoriatic Arthritis Uncluttered Screening Evaluation – PURE4 0), and no new outbreaks of BP.

**Fig. 4:**
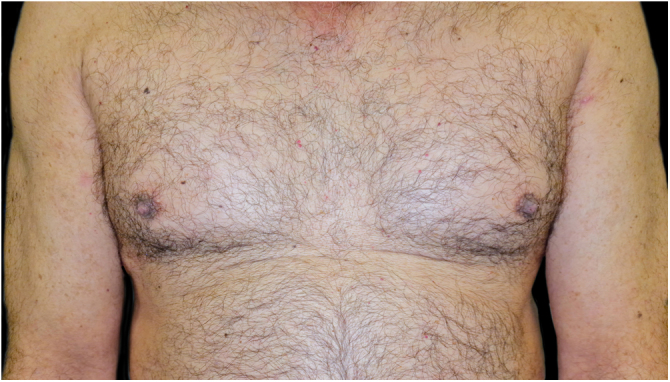
Complete resolution of Psoriasis Plaques (PASI0) and no new bullous pemphigoid lesions after 2-months with guselkumab.

The association between psoriasis and BP is described in the literature at the epidemiological level, as patients with BP are approximately 2.5 times more likely to develop psoriasis than the general population.^1^, ^2^, ^3^

It has been proposed that psoriasis may cause tissue damage that exposes certain antigens that trigger an autoimmune response.^3^ It has also been suggested that there could be an architectural alteration of the basement membrane found in psoriasis, both in the plaque itself and in apparently healthy skin. The most relevant findings are the disintegration and irregular distribution of both laminin and fibronectin 3, which lead to a weakening of the basement membrane that may facilitate its damage in case of intercurrence with BP.^4^

Furthermore, it seems that elevated levels of Interleukin (IL)-23 are not only elevated in psoriasis, but also in BP (whether or not associated with psoriasis). IL-23 could play a role in that it produces an overexpression of Matrix Metallopeptidase (MMP)-9, which weakens the basement membrane.^5^

There are currently no published cases of treatment of BP with anti-IL23 drugs, although there is one case treated with an anti-IL12/23 drug (ustekinumab).^6^ In contrast, there have been reported some cases of BP development associated with the initiation of anti-IL23 or anti-IL12/23 drugs, in which, interestingly, most of them did not present positivity for BP autoantibodies.^7^, ^8^, ^9^

The mechanism whereby guselkumab has been effective in our case remains unknown. It could be that the regulation of the IL-23 axis directly controls BP. Another possibility is that guselkumab improves psoriasis primarily, thus achieving a stabilization of the basement membrane that avoids the concurrence of BP.

The relation between anti-IL23 drugs and BP is not defined. However, there are data that support the biological plausibility that they may be effective in this pathology. Therefore, they could be considered in the future as a therapeutic alternative in multirefractory cases, as long as more studies are carried out.

## Financial support

None declared.

## Authors’ contributions

Francisco José Rodríguez-Cuadrado: Study concept and design; data collection, or analysis and interpretation of data; writing of the manuscript or critical review of important intellectual content; critical review of the literature; final approval of the final version of the manuscript.

Gaston Roustan-Gullón: Writing of the manuscript or critical review of important intellectual content; intellectual participation in the propaedeutic and/or therapeutic conduct of the studied cases; critical review of the literature; final approval of the final version of the manuscript.

Dolores Suárez-Massa: Writing of the manuscript or critical review of important intellectual content; intellectual participation in the propaedeutic and/or therapeutic conduct of the studied cases; critical review of the literature; final approval of the final version of the manuscript.

Mercedes Hospital-Gil: Writing of the manuscript or critical review of important intellectual content; intellectual participation in the propaedeutic and/or therapeutic conduct of the studied cases; critical review of the literature; final approval of the final version of the manuscript.

## Conflicts of interest

None declared.
